# ErbB2 and NFκB Overexpression as Predictors of Chemoradiation Resistance and Putative Targets to Overcome Resistance in Muscle-Invasive Bladder Cancer

**DOI:** 10.1371/journal.pone.0027616

**Published:** 2011-11-10

**Authors:** Fumitaka Koga, Soichiro Yoshida, Manabu Tatokoro, Satoru Kawakami, Yasuhisa Fujii, Jiro Kumagai, Len Neckers, Kazunori Kihara

**Affiliations:** 1 Department of Urology, Tokyo Medical and Dental University Graduate School, Tokyo, Japan; 2 Department of Surgical Pathology, Tokyo Medical and Dental University Graduate School, Tokyo, Japan; 3 Urologic Oncology Branch, National Cancer Institute, Bethesda, Maryland, United States of America; Instituto Nacional de Câncer, Brazil

## Abstract

Radical cystectomy for muscle-invasive bladder cancer (MIBC) patients frequently impairs their quality of life (QOL) due to urinary diversion. To improve their QOL, a bladder-sparing alternative strategy using chemoradiation has been developed. In bladder-sparing protocols, complete response (CR) to induction chemoradiation is a prerequisite for bladder preservation and favorable survival. Thus predicting chemoradiation resistance and overcoming it would increase individual MIBC patients' chances of bladder preservation. The aim of this study is to investigate putative molecular targets for treatment aimed at improving chemoradiation response. Expression levels of erbB2, NFκB, p53, and survivin were evaluated immunohistochemically in pretreatment biopsy samples from 35 MIBC patients in whom chemoradiation sensitivity had been pathologically evaluated in cystectomy specimens, and associations of these expression levels with chemoradiation sensitivity and cancer-specific survival (CSS) were investigated. Of the 35 patients, 11 (31%) achieved pathological CR, while tumors in the remaining 24 patients (69%) were chemoradiation-resistant. Multivariate analysis identified erbB2 and NFκB overexpression and hydronephrosis as significant and independent risk factors for chemoradiation resistance with respective relative risks of 11.8 (*P* = 0.014), 15.4 (*P* = 0.024) and 14.3 (*P* = 0.038). The chemoradiation resistance rate was 88.5% for tumors overexpressing erbB2 and/or NFκB, but only 11.1% for those negative for both (*P* <0.0001). The 5-year CSS rate was 74% overall. Through multivariate analysis, overexpression of erbB2 and/or NFκB was identified as an independent risk factor for bladder cancer death with marginal significance (hazard ratio 21.5, *P* = 0.056) along with chemoradiation resistance (*P* = 0.003) and hydronephrosis (*P* = 0.018). The 5-year CSS rate for the 11 patients achieving pathological CR was 100%, while that for the 24 with chemoradiation-resistant disease was 61% (*P* = 0.018). Thus, erbB2 and NFκB overexpression are relevant to chemoradiation resistance and are putative targets aimed at overcoming chemoradiation resistance in MIBC.

## Introduction

Bladder cancer is the 5^th^ most common cancer in the US, with 70,530 new patients and 14,680 deaths recorded in 2010 [1]. Muscle-invasive bladder cancer (MIBC) accounts for one-third of all bladder cancer cases [2]. Radical cystectomy is the reference standard of care for patients with MIBC, but urinary diversion, which must be performed concurrently with radical cystectomy, and postsurgical morbidity potentially compromise patients' quality of life. To overcome these issues, bladder-sparing approaches with various modalities have been investigated. Among them, trimodality protocols consisting of transurethral resection (TUR), chemotherapy, and radiotherapy have yielded the most favorable oncological outcomes, with 5-year overall survival rates of 50 to 60%, which are comparable to contemporary survival rates of immediate radical cystectomy series [3], [4]. In most chemoradiotherapy (CRT)-based bladder-sparing protocols, patients who achieve complete response (CR) after induction CRT are selectively subjected to consolidative therapies for bladder preservation, whereas those who do not achieve CR are recommended to undergo radical cystectomy [5]. In addition, the response to induction CRT has a strong impact on the survival of MIBC patients regardless of subsequent radical cystectomy with curative intent [6], [7]. Thus, the ability to predict non-CR patients and the development of a novel strategy to improve their response to CRT would increase the chances of bladder preservation and potentially improve survival outcomes in MIBC patients.

A number of translational studies have been conducted to identify molecular markers that predict therapeutic response to chemotherapy and/or radiotherapy in patients with MIBC or more advanced forms of the disease. Several molecules and genetic modifications have been identified as predictors of CRT response in MIBC [8], [9], [10], [11], [12]. Yet most studies have focused on predicting CRT response without also specifically attempting to improve CRT response by targeting the molecules responsible for CRT resistance.

The aim of the current study is to identify putative molecular targets to improve CRT sensitivity and thereby to improve patient prognosis. Of several molecular markers reportedly predicting unfavorable tumor response to CRT or radiotherapy [8], [13], [14], [15], [16], [17], [18], [19], [20], those that can be targeted by small molecules or monoclonal antibodies include erythroblastic leukemia viral oncogene homolog 2 (erbB2, also known as HER2/neu) [8], nuclear factor-κB (NFκB) [13], [14], mutant p53 [17], [18], and survivin [19]. We investigated associations of the expression profiles of these oncoproteins with both CRT sensitivity and cancer-specific survival (CSS) in a cohort of MIBC patients in whom CRT sensitivity of tumors had been pathologically evaluated in cystectomy specimens.

## Methods

### Ethical statement

The ethical committee of Tokyo Medical and Dental University reviewed and approved the current study protocol (approval number 602). Written informed consent was obtained from all patients whose tumor tissues were examined for expression of CRT resistance-associated proteins.

### Patients

Between 1997 and 2008, 162 MIBC patients without clinical metastasis were treated in accordance with our institutional therapeutic protocol, which is described elsewhere [7], [21], [22]. Briefly, patients underwent transurethral resection (TUR) of bladder tumor and induction CRT consisting of external beam radiotherapy to the small pelvis (40 Gy) and two cycles of concurrent intravenous cisplatin (20 mg/day for 5 days) separated by a three-week interval. When bladder cancer was intravesically localized and did not involve the bladder neck and trigone, and residual disease was clinically absent or present only in a small quantity after induction CRT, the patient could selectively undergo consolidative partial cystectomy with pelvic lymph node dissection for bladder preservation; otherwise radical cystectomy was recommended. Of the 162 patients, 88 who underwent visibly complete TUR before CRT and another 39 who did not undergo cystectomy were excluded. In the remaining 35 patients in whom tumor sensitivity to CRT was assessed pathologically in cystectomy specimens, we investigated clinical and molecular variables relevant to CRT resistance, which was defined as the presence of pathological residual disease (non-CR) in cystectomy specimens.

### TNM stage

Clinical stage was determined according to the 2002 TNM system [23] based on findings obtained through TUR, bimanual examination and CT of the chest, abdomen and pelvis. MRI of the pelvis was also included starting in 2007.

### Follow-up

Patients were followed essentially at 3-month intervals for the first 2 years, at 6-month intervals for the following 3 years, and yearly thereafter. Evaluations consisted of medical history, physical examination, complete blood counts and blood chemistry, and CT of the chest, abdomen and pelvis. Urine cytology, cystoscopy and pelvic MRI were added for bladder-spared patients. Survival intervals were calculated from the first day of CRT to the date of death.

### Immunohistochemistry

Immunostaining was performed as previously described [24] using formalin-fixed paraffin-embedded tumor tissues obtained at TUR before induction CRT. Primary antibodies used were pre-diluted rabbit polyclonal anti-erbB2 antibody (Dako Cytomation, Glostrup, Denmark; at 1:300), rabbit monoclonal antibody against survivin (Cell Signaling Technology, Danvers, MA, USA; at 1:200), mouse monoclonal antibody against p53 (Pab1801; at 1:40) and NFκB p65 (F-6; Santa Cruz Biotechnology, Santa Cruz, CA, USA; at 1:100). Percentages of tumor cells positive for immunostaining were scored. Since NFκB p65 exerts its oncogenic properties as a nuclear transcription factor, nuclear immunoreactivity was evaluated as positive. The slides were reviewed at a magnification of ×200 by two independent observers (Y.S. and J.K.) who were blinded to clinicopathological data. In cases of observer disagreement, a final decision was made by consensus between the two observers.

### Statistical Analysis

Differences between groups were assessed using the Chi-square test for categorical data. Cut-off values of continuous variables best predicting CRT resistance were determined by partition analysis. Risk factors for CRT resistance and bladder cancer death were evaluated using logistic regression analysis and the Cox proportional hazard model, respectively. In multivariate analysis, variables were selected using the backward stepwise method. Survival curves were plotted using the Kaplan-Meier method and their differences were assessed by log-rank test. These statistical analyses were performed using JMP 8.0 statistical software (SAS Institute, Cary, NC, USA). Differences were considered significant at *P <*0.05.

## Results

Baseline statistics of the 35 eligible patients are listed in Table 1. All MIBCs comprised high-grade urothelial carcinomas. Four (11%) underwent partial cystectomy as consolidative therapy for bladder preservation while 31 (89%) underwent radical cystectomy. Of the 35 patients, 11 (31%) achieved pathological CR; of the remaining 24 patients with CRT-resistant MIBC, pathological T stages were Tis in 2 (6%), T1 in 8 (23%), T2 in 4 (11%), T3 in 9 (26%), and T4a in 1 (3%). Pathological lymph node metastasis was found in 3 patients (9%).

**Table 1:** Baseline statistics of the 35 MIBC patients who underwent induction CRT followed by cystectomy.

Variables	No. of patients (%)
Gender	
Female	15 (43)
Male	20 (57)
Median (range) age, years	71 (45-83)
Clinical T stage	
T2	14 (40)
T3	18 (51)
T4a	3 (9)
Tumor size	
<5 cm	17 (49)
≥5 cm	18 (51)
Multifocality	
No	12 (34)
Yes	23 (66)
Presence of CIS	
No	28 (80)
Yes	7 (20)
Presence of hydronephrosis	
No	23 (66)
Yes	12 (34)
Pathological T stage of cystectomy specimens	
T0	11 (31)
Tis/1/2	2 (6)/8 (23)/4 (11)
T3/4a	9 (26)/1 (3)
Pathological N stage	
N0	32 (91)
N1/2	2 (6)/1 (3)
ErbB2	
Negative (<20%)	15 (43)
Positive (≥20%)	20 (57)
NFκB	
Negative (<20%)	23 (66)
Positive (≥20%)	12 (34)
p53	
Negative (<10%)	10 (29)
Positive (≥10%)	25 (71)
Survivin	
Negative (<20%)	7 (20)
Positive (≥20%)	28 (80)

**Figure 1:**
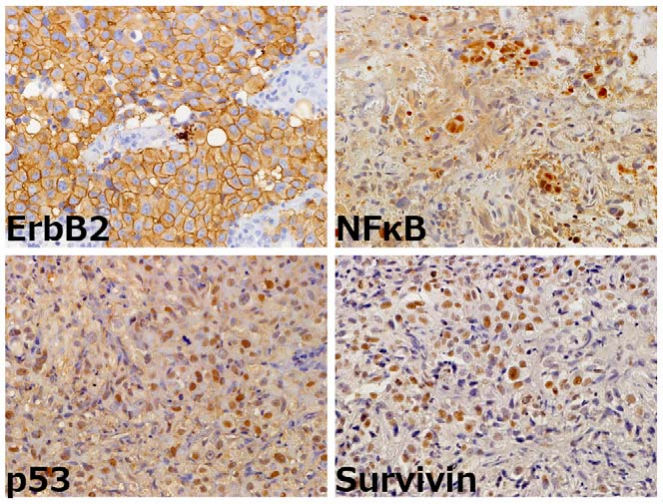
Representative microphotographs of MIBC positive for erbB2 (upper left), NFκB (upper right), p53 (lower left), and survivin (lower right). Reduced from ×400.

Immunoreactivity for erbB2, NFκB, p53, and survivin was observed in 20 (57%), 16 (46%), 25 (71%), and 28 patients (80%), respectively (Figure 1). The cut-off value of immunoreactivity that best predicted clinical CRT resistance was 20% for erbB2, NFκB, and survivin. For p53, a previously reported cut-off value of 10% was used [25].

Among the clinical and molecular variables listed in Table 1, univariate analysis revealed that erbB2 overexpression was significantly associated with CRT resistance (*P* = 0.003, Table 2); NFκB overexpression, the presence of hydronephrosis, and tumor size ≥5 cm were also associated with CRT resistance despite not reaching statistical significance (*P* = 0.055, 0.056, and 0.075, respectively). Multivariate analysis identified erbB2 and NFκB overexpression and the presence of hydronephrosis as significant and independent risk factors for CRT resistance, with respective relative risks of 11.8 (*P* = 0.014), 15.4 (*P* = 0.024) and 14.3 (*P* = 0.038, Table 2). Neither p53 nor survivin was significantly associated with CRT resistance. Ability to predict CRT resistance was strengthened when data on the expression of both erbB2 and NFκB were used; the CRT resistance rate was 88.5% among tumors overexpressing erbB2 and/or NFκB but only 11.1% among those negative for both (*P* <0.0001). Thus, erbB2 and NFκB overexpression are relevant to CRT resistance in MIBC.

**Table 2:** Risk factors predicting CRT resistance.

Variables	Univariate		Multivariate	
	CRT resistance rate (%)	*P* value	RR	*P* value
Gender		0.28		0.062
Female	80.0		7.79	
Male	60.0		1	
Age		0.17		
<71	81.3			
≥71	57.9			
Clinical T stage		0.28		
T2	57.1			
T3-4a	76.2			
Tumor size		0.075		
<5 cm	52.9			
≥5 cm	83.3			
Multifocality		0.71		
No	75.0			
Yes	65.2			
Presence of CIS		1.00		
No	67.9			
Yes	71.4			
Presence of hydronephrosis		0.056		0.038
No	56.5		1	
Yes	91.7		14.3	
ErbB2		0.003		0.014
Negative (<20%)	46.7		1	
Positive (≥20%)	85.0		11.8	
NFκB		0.055		0.024
Negative (<20%)	56.5		1	
Positive (≥20%)	91.7		15.4	
p53		0.45		
Negative (<10%)	80.0			
Positive (≥10%)	64.0			
Survivin		1.00		
Negative (<20%)	71.4			
Positive (≥20%)	67.9			
Combination of erbB2 and NFκB		<0.0001		-
Dual negative	11.1			
Single or dual positive	88.5			

RR, relative risk.

During the follow-up period (median 60 months, range 19-104), 10 patients died of bladder cancer. The 5-year CSS rate was 74%. Clinicopathological and molecular variables associated with bladder cancer death are listed in Table 3. Univariate analysis revealed that CRT resistance and the presence of hydronephrosis were significantly associated with bladder cancer death (*P* = 0.003 and 0.008, respectively). Multivariate analysis revealed that CRT resistance was the strongest and most independent risk factor (*P* = 0.003) followed by the presence of hydronephrosis (*P* = 0.018). Overexpression of ErbB2 and/or NFκB was also identified as an independent risk factor for bladder cancer death with marginal statistical significance (*P* = 0.056). The 5-year CSS rate among the 11 patients with pathological CR was 100%, while that for the remaining 24 with CRT-resistant MIBC was 61% (*P* = 0.018). Thus high sensitivity to CRT is associated with excellent survival outcomes among MIBC patients.

**Table 3:** Risk factors predicting bladder cancer death.

	Univariate		Multivariate	
Variables	HR	*P* value	HR	*P* value
Gender		0.94		
Female vs Male	1.05			
Age		0.80		
<71 vs ≥71	1.18			
Clinical T stage		0.057		
T3-4a vs 2	3.91			
Tumor size		0.37		
≥5 cm vs <5 cm	1.78			
Multifocality		0.20		
Yes vs No	2.54			
Presence of CIS		0.32		
Yes vs No	0.40			
Presence of hydronephrosis		0.008		0.018
Yes vs No	5.71		5.56	
ErbB2		0.97		
Positive vs Negative	1.03			
NFκB		0.24		
Positive vs Negative	2.13			
Combination of erbB2 and NFκB		0.19		0.056
Single or dual positivevs dual negative	3.26		21.5	
p53		0.53		
Positive vs Negative	0.64			
Survivin		0.30		
Positive vs Negative	0.46			
CRT resistance		0.003		0.003
Yes vs No	6.9×10^7^		5.2×10^6^	

HR, hazard ratio.

## Discussion

The current study showed that erbB2 and NFκB overexpression play a potential role in CRT resistance and are independently associated with unfavorable CSS with marginal significance in MIBC patients treated with induction CRT plus cystectomy. This indicates that erbB2 and NFκB are putative therapeutic targets for treatments aimed at improving CRT sensitivity in MIBC. This is the first study to demonstrate the relationship between NFκB overexpression and CRT resistance in MIBC and the potential prognostic impact of a combined expression profile of erbB2 and NFκB in patients treated with induction CRT and cystectomy. As we and others have demonstrated, a better response to CRT is associated with more favorable survival outcomes [4], [7], [26]. Thus, enhancing CRT sensitivity by targeting erbB2 and NFκB may improve survival times among patients treated with CRT.

ErbB2 is a member of the epidermal growth factor receptor (EGFR) family of receptor tyrosine kinases, which generate a complex program of signal transduction events that modulates cell proliferation, differentiation, invasion, and survival [27]. Unlike EGFR, erbB2 does not have its own ligand; instead, it heterodimerizes with other activated EGFR members to enhance and prolong their signaling. In cancer cells overexpressing erbB2 due to gene amplification, high basal autophosphorylation of erbB2 promotes oncogenic activities including metastasis and therapeutic resistance [27]. According to previous reports on MIBC, erbB2 overexpression is an independent risk factor for cancer death in patients undergoing radical cystectomy [28] and an independent predictor of non-CR after CRT but not a risk factor for cancer death in patients treated with CRT [8]. The results of our study are consistent with those of the latter study, theoretically supporting the adjunctive use of erbB2 inhibitors to improve CRT sensitivity.

NFκB is a transcription factor involved in cellular response to various extracellular stimuli such as stress and cytokines [29]. In cancer cells, NFκB is often activated aberrantly, promoting the invasion, metastasis, and survival of these cells. Indeed, NFκB overexpression is associated with poor prognosis in various malignancies including MIBC [30] and with CRT resistance in esophageal cancer [13], [14]. The current study showed that NFκB overexpression is closely related to CRT resistance in MIBC and that, in cooperation with erbB2 overexpression, it potentially affects the prognoses of MIBC patients treated with induction CRT and cystectomy. These results strengthen the case for a putative role of NFκB inhibitors, of which many types are under clinical investigation [29], in overcoming CRT resistance in MIBC overexpressing NFκB.

The current study indicates that both erbB2 and NFκB are putative targets for treatments to overcome CRT resistance in MIBC. Among the small molecules currently being investigated in clinical studies, heat shock protein 90 (Hsp90) inhibitors can simultaneously block the activity of both of these oncoproteins [31]. Hsp90 is a ubiquitous molecular chaperone required for the stability and function of numerous client proteins, including a number of oncoproteins essential for the acquisition and maintenance of cancer hallmarks [31], [32], [33]. Hsp90 inhibitors destabilize Hsp90 clients by dissociating Hsp90-Hsp90 client complexes and thereby exerting antitumor activity [34]. Since both erbB2 and inhibitor of κB kinase β (IKKβ), which mediates NFκB activation, are Hsp90 client proteins [31], adjunctive use of Hsp90 inhibitors is expected to efficiently sensitize MIBCs overexpressing erbB2 and/or NFκB to CRT. Indeed, we have confirmed that low-dose Hsp90 inhibitors effectually potentiate the effects of CRT on bladder cancer cells overexpressing erbB2 and NFκB in preclinical models [35].

In most bladder-sparing protocols for MIBC, CR after induction CRT is a prerequisite for bladder preservation; patients with residual disease are at higher risk for bladder cancer death despite undergoing salvage radical cystectomy [4], [7], [26]. This is the reason why CRT resistance was stringently defined as pathological non-CR in the current study. When CRT resistance was instead defined as the presence of disease at pathological stage T2 or greater remaining in a cystectomy specimen, overexpression of survivin (with a cut-off value of 40%) was also identified as an independent predictor of CRT resistance. Survivin, a member of the inhibitor of apoptosis protein family, is reportedly associated with local failure in high-grade T1 bladder cancer patients treated with CRT [19]. Recently, a small molecule inhibiting survivin has been identified and is now in clinical trials [36]. This compound is also expected to sensitize MIBC to CRT in clinical settings.

The current study has some limitations including a small cohort size, selection bias, and its retrospective nature. To precisely assess CRT sensitivity in cystectomy specimens from patients who had visible tumors before CRT, only 35 out of 162 patients were eligible for analysis. The fact that statistically significant results were obtained despite such a small number of subjects might be attributed to the stringent selection of subjects for analysis. A prospective study in a larger cohort is needed to validate the findings obtained in the current retrospective study.

In conclusion, erbB2 and NFκB overexpression are relevant to CRT resistance of MIBC, and overexpression of at least one of these oncoproteins potentially increases the risk for cancer death in MIBC patients treated with induction CRT and cystectomy. ErbB2 and NFκB are potential targets for treatment to overcome CRT resistance of MIBC.
